# Proportional Upregulation of CD97 Isoforms in Glioblastoma and Glioblastoma-Derived Brain Tumor Initiating Cells

**DOI:** 10.1371/journal.pone.0111532

**Published:** 2015-02-25

**Authors:** Michael Safaee, Shayan Fakurnejad, Orin Bloch, Aaron J. Clark, Michael E. Ivan, Matthew Z. Sun, Taemin Oh, Joanna J. Phillips, Andrew T. Parsa

**Affiliations:** 1 Department of Neurological Surgery, University of California San Francisco, San Francisco, California, United States of America; 2 Department of Neurological Surgery, Northwestern University Feinberg School of Medicine, Chicago, Illinois, United States of America; University of Michigan School of Medicine, UNITED STATES

## Abstract

CD97 is a novel glioma antigen that confers an invasive phenotype and poor survival in patients with glioblastoma (GBM), the most aggressive primary malignant brain tumor. The short isoform of CD97, known as EGF(1,2,5), has been shown to promote invasion and metastasis, but its role in gliomas and GBM-derived brain tumor initiating cells (BTICs) has not been studied. We sought to characterize CD97 expression among gliomas and identify the specific isoforms expressed. The short isoform of CD97 was identified in GBM and GBM-derived BTICs, but not low grade or anaplastic astrocytomas. All samples expressing the EGF(1,2,5) isoform were also found to express the EGF(1,2,3,5) isoform. These isoforms are believed to possess similar ligand binding patterns and interact with chondroitin sulfate, a component of the extracellular matrix, and the integrin α5β1. Using data acquired from the Cancer Genome Atlas (TCGA), we show that CD97 is upregulated among the classical and mesenchymal subtypes of GBM and significantly decreased among IDH1 mutant GBMs. Given its proven roles in tumor invasion, expression among aggressive genetic subtypes of GBM, and association with overall survival, CD97 is an attractive therapeutic target for patients with GBM.

## Introduction

Glioblastoma (GBM) is the most common and aggressive primary malignant brain tumor with a median survival of less than two years [1,2]. The invasive nature of these tumors prevents complete removal despite aggressive surgical resection. Intracranial dissemination, either at diagnosis or progression, is a poor prognostic factor associated with decreased survival [3]. Mechanisms underlying glioma invasion are complex and only partially understood. We have previously demonstrated that both U251 and U87MG GBM cell lines robustly express CD97. When CD97 expression was suppressed using small interfering RNA (siRNA), these cell lines demonstrated significant reductions in migration and invasion with no effect on proliferation [4].

CD97 is a member of the epidermal growth factor seven-span transmembrane (EGF-TM7) family of adhesion G-protein coupled receptors (GPCRs), which consists of proteins that are expressed mainly on the surface of leukocytes [5]. CD97 is found on lymphocytes, monocytes, macrophages, dendritic cells, granulocytes, and smooth muscle [6]. It is rapidly upregulated during lymphocyte activation and has been implicated in cell adhesion and migration via interactions with cell surface proteins and components of the extracellular matrix (ECM). The three known ligands of CD97 include: CD55, a negative regulator of the complement cascade [7], chondroitin sulfate, a component of the ECM [8–10], and the integrin α5β1 [11]. The association with integrins is particularly noteworthy since they have been shown to mediate invasion, migration, and angiogenesis in GBM [12,13]. Proliferating endothelial cells in the brain are known to express chondroitin sulfate, suggesting a potential interaction between tumor cells and nascent vasculature [14].

CD97 is expressed in a variety of malignancies including thyroid, gastric, esophageal, pancreatic, and colorectal cancers [15–17]. It has been shown to correlate with both lymph node invasion [16] and poor clinical staging [15], with expression generally highest at the tumor’s invasive front or leading edge [15,17,18]. Functionally, CD97 has been shown to confer an invasive phenotype and stimulate angiogenesis [11,19]. CD97 has been described in GBM [20] and our group recently demonstrated an association with invasion and migration [4]. We also demonstrated, for the first time, an association between increased CD97 expression and poor survival in GBM patients. GBMs are characterized by their more aggressive behavior compared to other low grade gliomas, however the expression of CD97 across histologic grades has not been studied. The expression of CD97 in GBM-derived brain tumor initiating cells is also unknown. Brain tumor initiating cells, also referred to as glioma stem cells, are characterized by the expression of a number of stem cell markers and possess the capacity for both self-renewal and differentiation into tumors; they are implicated in chemoresistance [21], radioresistance [22], angiogenesis [23], and tumor recurrence [24–26]. In this study we sought to characterize the expression of CD97 across glioma grades and within GBM-derived brain tumor initiating cells.

## Methods

### Cell culture

All research activities were approved by the University of California, San Francisco Committee on Human Research (UCSF CHR), our institutional review board for human research, with both written and verbal consent provided from patients. Brain tumor initiating cell lines were established from surgical specimens acquired at our institution. Freshly resected tumors, pathologically confirmed as GBM, were dissociated into single cells using Papain at 20 units/ml (Worthington Biochemical Corporation) for 20 minutes at 37°C. The dissociated tissue was filtered through a 70 μM cell strainer to remove excess debris and the remaining cells suspension plated in serum-free media supplemented with N2, B27, EGF, and FGF (20 ng/ml) as previously described [27,28]. Culture vessels were coated with Laminin (Sigma) for 3 hr at 10 ug/ml prior to use. Media was changed every 3 days until cells were confluent at which point they were detached using Accutase (Sigma) then split 1:3 to 1:5.

### Analysis of frozen tissue

Flash frozen glioma specimens were acquired from the UCSF Brain Tumor Research Center. Samples included pathologically confirmed GBMs (WHO grade IV), anaplastic astrocytomas (WHO grade III), and low grade gliomas (WHO grade II). Tissue specimens were homogenized using a mortar and pestle to extract RNA and protein. For isolation of protein, homogenate was resuspended in 1x RIPA buffer (Cell Signaling) and placed on a rotating rack for 1 hour at 4°C. Insoluble cell fragments were removed by centrifugation at 13,000 rpm for 20 min. Protein was then quantified by BCA assay (Thermo Scientific) and analyzed by Western blot.

### Immunoblotting

At 75–80% confluence, cells were treated with 150 μl of 10x lysis buffer (Cell Signaling Technology) combined with protease inhibitor tablets (Roche), phosphatase inhibitor tablets (Roche), and 1 mM phenylmethanesulfonyl fluoride (PMSF). Samples were run on a 4–20% Tris-Glycine gel (Invitrogen) and transferred to an Immun-Blot PVDF membrane (BioRad). After blocking in 5% nonfat dry milk (BioRad), samples were incubated in the following primary antibodies: anti-CD97 1:500 (Abcam ab108368), anti-nestin 1:4,000 (abcam ab22035), anti-Sox2 1:1,000 (Abcam ab75485), anti-GAPDH 1:10,000 (Cell Signaling 2118S). Secondary antibodies conjugated to horseradish peroxidase were used with the Amersham ECL system (GE Healthcare) to detect protein.

### Immunocytochemistry

Cells were plated on 12 mm cover glass at a density of 2.5 x 10^5^ cells/cover glass (Fisher Scientific), then cultured for 2 days until reaching 80–90% confluence. Cells were fixed with 4% formaldehyde at room temperature, permeabilized with cold methanol, then blocked in phosphate buffered saline (PBS) with 5% fetal bovine serum (FBS), 2 mg/ml bovine serum albumin (BSA), and 0.1% Triton X-100 for 1 hour at room temperature. Slides were incubated with the following primary antibodies: anti-CD97 1:100 (Abcam ab108368), anti-α-tubulin 1:100 (Cell Signaling 2125S) for 1 hour at room temperature, followed by secondary antibody at 1:250 conjugated to Alexa Fluor 488 (Invitrogen) for 1 hour. Specimens were mounted using a DAPI-containing mounting medium (Vector Labs). Confocal images were generated on a Zeiss LSM 510 META laser-scanning microscope.

### Quantitative RT-PCR

Total mRNA was isolated using the QIAGEN RNeasy Micro Kit (QIAGEN). cDNA was generated using the RT^2^ First Strand Kit (QIAGEN) with quantitative PCR performed using Power SYBR Green PCR mix (Applied Biosystems) on a CFX96 Real-Time System (BioRad). All samples were repeated in triplicate and normalized to 18S rRNA. Separate primers were used to amplify CD97 isoforms, which were visualized on a 2% agarose gel. Primers were designed using Primer 3 software (MIT): CD97(total)-F, GCTTGGTGCTGACCTATGTG; CD97(total)-R, GGTCTGATTGTGGCCAGTG; CD97(EGF1,2,5)-F, ACTCTGCCGGGAGCTGAAAC; CD97(EGF1,2,5)-R, TGGATGGTGACCTCGGCTGA; CD97(EGF1,2,3,5)-F, AAGGCTCTGTAAAAGCTACG; CD97(EGF1,2,3,5)-R, TCACAGACAGTGTCCTTTTG; 18S rRNA-F, GTAACCCGTTGAACCCCATT; 18S rRNA-R, CCATCCAATCGGTAGTAGCG. For isoform-specific quantification, RASE primer design, as described by Brosseau et al.[29], was used to generate the following: CD97(EGF1,2,3,5)-F, TGGCCCAAACAATACCGTCTGT; EGF(1,2,3,5)-R, TGAACCCACGGTGTTGAAGCA; β-actin-F, GATGAGATTGGCATGGCTTT; β-actin-R, CACCTTCACCGTTCCAGTTT.

### PCR product extraction and sequencing

DNA was extracted from agarose gels using the QIAquick Gel Extraction Kit (QIAGEN). PCR products were sequenced at the UCSF Genomics Core Facility using an Applied Biosystems 3730xl DNA Analyzer. Sequences were verified using the Genome Browser Gateway (UCSC).

### Analysis of The Cancer Genome Atlas (TCGA) Data

TCGA data was downloaded from the cBioPortal for Cancer Genomics (http://www.cbioportal.org/public-portal/) on March 10, 2013. CD97 expression z-scores, genomic subtype classification, and IDH1 mutation status were collected. For all statistical analysis performed in this study, continuous variables were compared using analysis of variance (ANOVA) with Tukey’s post-hoc test or independent samples t test. Dichotomous variables compared using the Chi-square test.

## Results

### Characterization of isoforms in GBM cell lines

**Figure 1:**
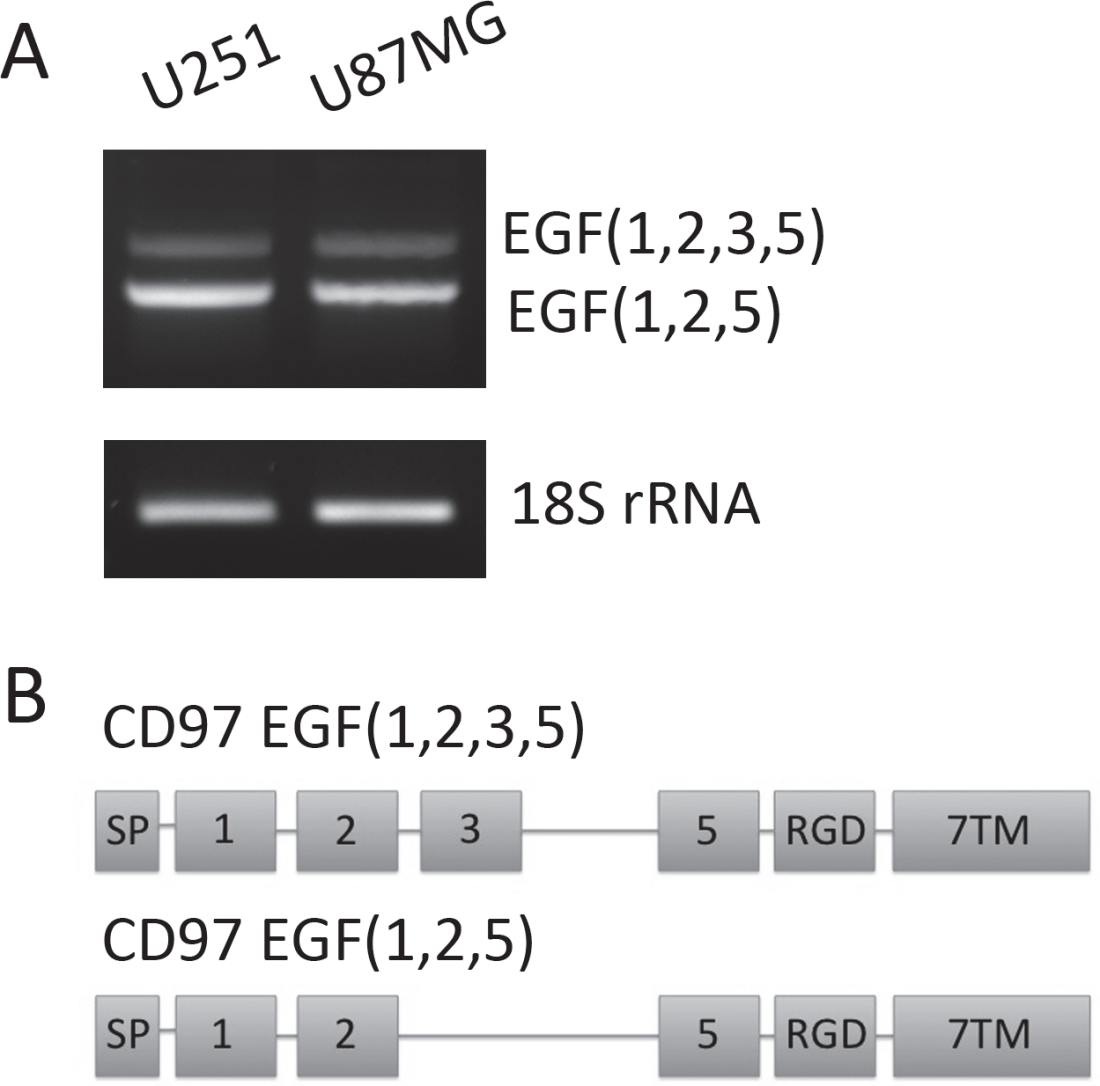
Characterization of CD97 isoforms in glioblastoma. RNA was isolated from the GBM cell lines U251 and U87MG, then converted to cDNA. PCR with primers spanning the variably spliced regions of CD97 were used to identify the specific isoforms expressed in these cells. Sequencing analysis confirmed these as the EGF(1,2,5) and EGF(1,2,3,5) isoforms of CD97 (A). A schematic of these gene transcripts is also shown with the signal peptide (SP), RGD domain, and seven-span transmembrane (7TM) segments (B).

CD97 contains 5 extracellular EGF-like domains that are variably spliced to produce unique isoforms. To identify which isoforms are present in these cell lines, RNA was extracted and converted to cDNA. Primers were designed to span variably spliced regions, namely the second and fifth EGF domain (Fig. 1A). PCR products were run on a 2% agarose gel (Fig. 1B) and led to the identification of two unique transcripts. These PCR products were extracted and sequenced to confirm the identity of these isoforms. Both U251 and U87MG express the EGF(1,2,5) and EGF(1,2,3,5) isoforms of CD97 (Fig. 1C).

### Characterization of CD97 across glioma grades

**Figure 2:**
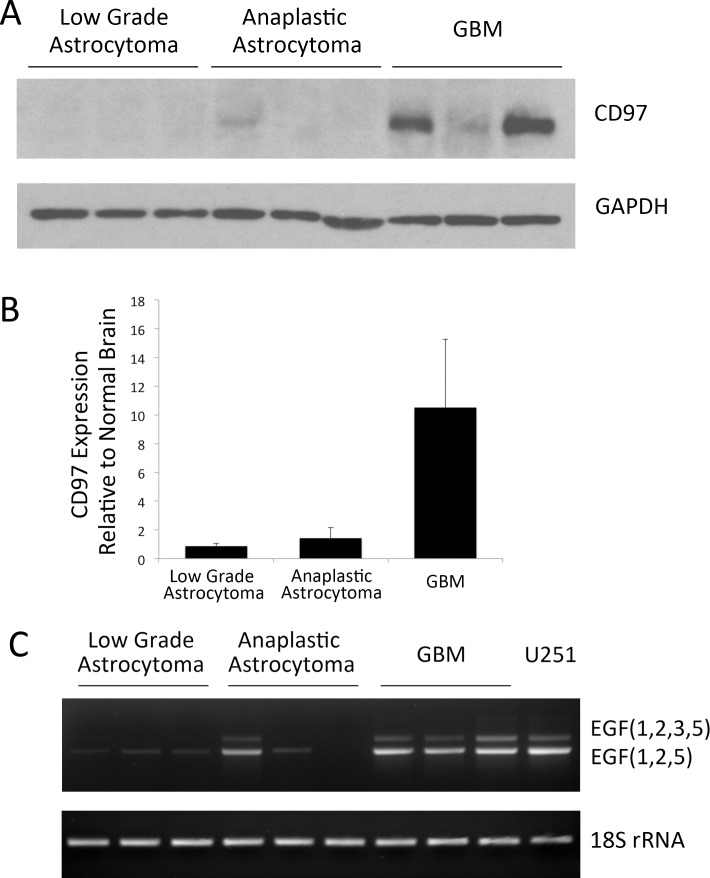
Expression of CD97 across glioma grades. Frozen tumor specimens histologically confirmed as low grade astrocytoma (n = 3), anaplastic astrocytoma (n = 3), and GBM (n = 3) were homogenized analyzed by Western blot. CD97 was expressed in the GBM specimens, but not low grade or anaplastic astrocytomas (A). Quantitative PCR was performed using these specimens and demonstrated a significant increase in CD97 expression among GBMs compared to low grade and anaplastic astrocytomas when normalized to normal brain (B). PCR using primers flanking the variably spliced regions of the CD97 transcript demonstrate the EGF(1,2,5) and EGF(1,2,3,5) in all GBM specimens and a single anaplastic astrocytoma, but not in the low grade or remaining anaplastic astrocytomas (C).

Although known to be expressed in GBM, expression of CD97 across lower grade gliomas has not been characterized. Nine frozen tumor specimens were homogenized and assessed for both mRNA and protein expression of CD97. On Western blot, GBM specimens were found to express CD97, while both low grade and anaplastic astrocytomas had no detectable CD97 protein (Fig. 2A). Using a quantitative PCR protocol, GBM samples had expression levels that were 10.5, 8.3, and 4.8-fold higher than normal brain homogenates (Fig. 2B). The fold-change in expression among low grade astrocytomas (WHO grade II) was 0.9, 0.3, and 0.2 relative to normal brain and 1.4, 1.3, and 0.7 relative to anaplastic astrocytomas (WHO grade III). In total, GBM specimens had an average 10-fold increase in CD97 transcript compared to normal brain, while both low grade astrocytoma and anaplastic astrocytoma samples had CD97 transcript levels that were essentially similar to normal brain (Fig. 2B).

**Figure 3:**
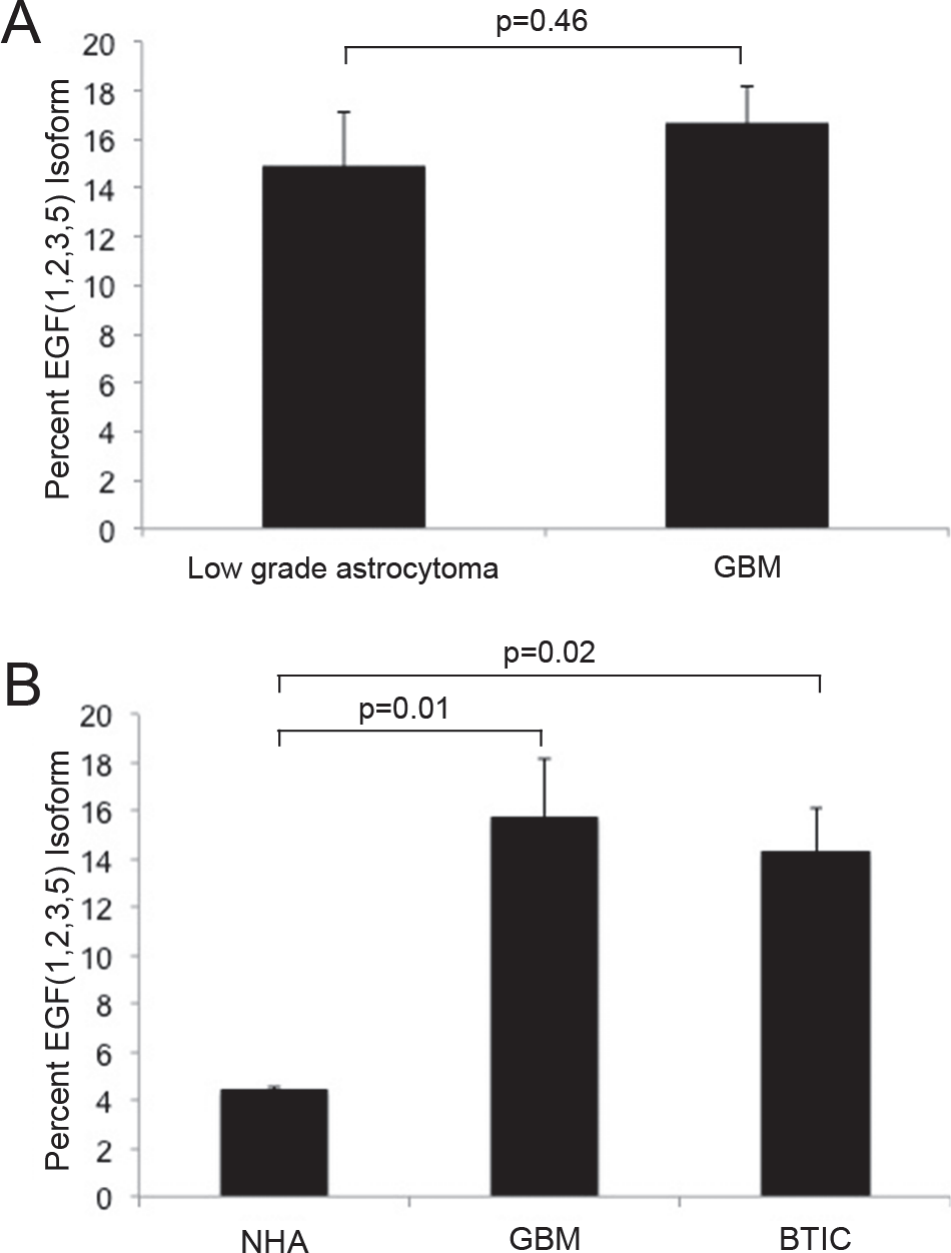
CD97 isoform expression in low grade astrocytoma and glioblastoma. Quantitative PCR was performed on frozen tumor specimens histologically confirmed as low grade astrocytoma (n = 3) and GBM (n = 3). The mean proportion of EGF(1,2,3,5) in low grade astrocytomas was 15% compared to 17% in GBM (p = 0.46), a difference that was not statistically significant (A). Among in vitro samples, the mean percent of EGF(1,2,3,5) comprising total CD97 in patient-matched GBM and BTIC cell lines was 16% and 14%, respectively (p = 0.83). Normal human astrocytes were found to express 4% EGF(1,2,3,5), which was significantly less than both GBM (p = 0.01) and BTIC (p = 0.02) cell lines (B).

To determine the isoforms of CD97 expressed in these specimens, we used PCR primers designed to span EGF domains 2 through 5. Similar to established GBM cell lines, all GBM samples expressed both EGF(1,2,5) and EGF(1,2,3,5) isoforms (Fig. 2C). One anaplastic astrocytoma expressed both EGF(1,2,5) and EGF(1,2,3,5), another expressed EGF(1,2,5), and a third expressed neither (Fig. 2C). Among low grade astrocytomas, there was faint presence of the EGF(1,2,5) isoform that appeared significantly less than the GBM samples (Fig. 2C). Quantification of CD97 isoforms was assessed by comparing levels of EGF(1,2,3,5) to total CD97 transcript (EGF(1,2,5) and EGF(1,2,3,5) combined). An isoform-specific primer that binds the exon junction corresponding to the third and fifth EGF domains was used to quantify the EGF(1,2,3,5) isoform. Primers that bind a common region of the CD97 transmembrane domain were used to quantify total levels of CD97 since they capture both isoforms. Among frozen tumor specimens, the average percentage of EGF(1,2,3,5) was 15 ± 2% among low grade astrocytoma specimens compared to 17 ± 2% among GBM specimens (Fig. 3A), a difference that was not statistically significant (p = 0.46). To determine if there was a difference in CD97 isoform expression among patient-matched GBMs and their associated BTICs, analysis was performed on specimens acquired from patients treated at our institution. Three patient-matched GBM and BTIC cell lines were found to express 16 ± 2% and 14 ± 2% of the EGF(1,2,3,5) isoform, respectively, compared to 4 ± 0.1% among normal human astrocytes (Fig. 3B), a difference that reached statistical significance (p = 0.008). Post-hoc analysis showed that the difference between GBM and BTIC was non-significant (p = 0.83), while both GBMs and BTICs expressed significantly higher levels of EGF(1,2,3,5) compared to normal human astrocytes (p = 0.01 and p = 0.02, respectively).

### CD97 expression in glioblastoma subtypes

**Figure 4:**
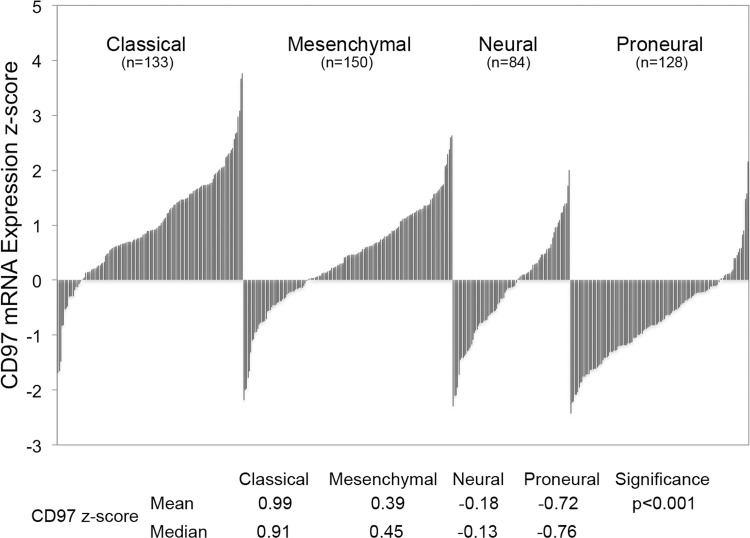
CD97 expression across genetic subtypes of glioblastoma. Gene expression data from the Cancer Genome Atlas (TCGA) was used to characterize CD97 expression across genetic subtypes of glioblastoma. CD97 upregulation was most commonly found in the classical and mesenchymal subtypes, while downregulation was more common in the neural and proneural subtypes.

**Figure 5:**
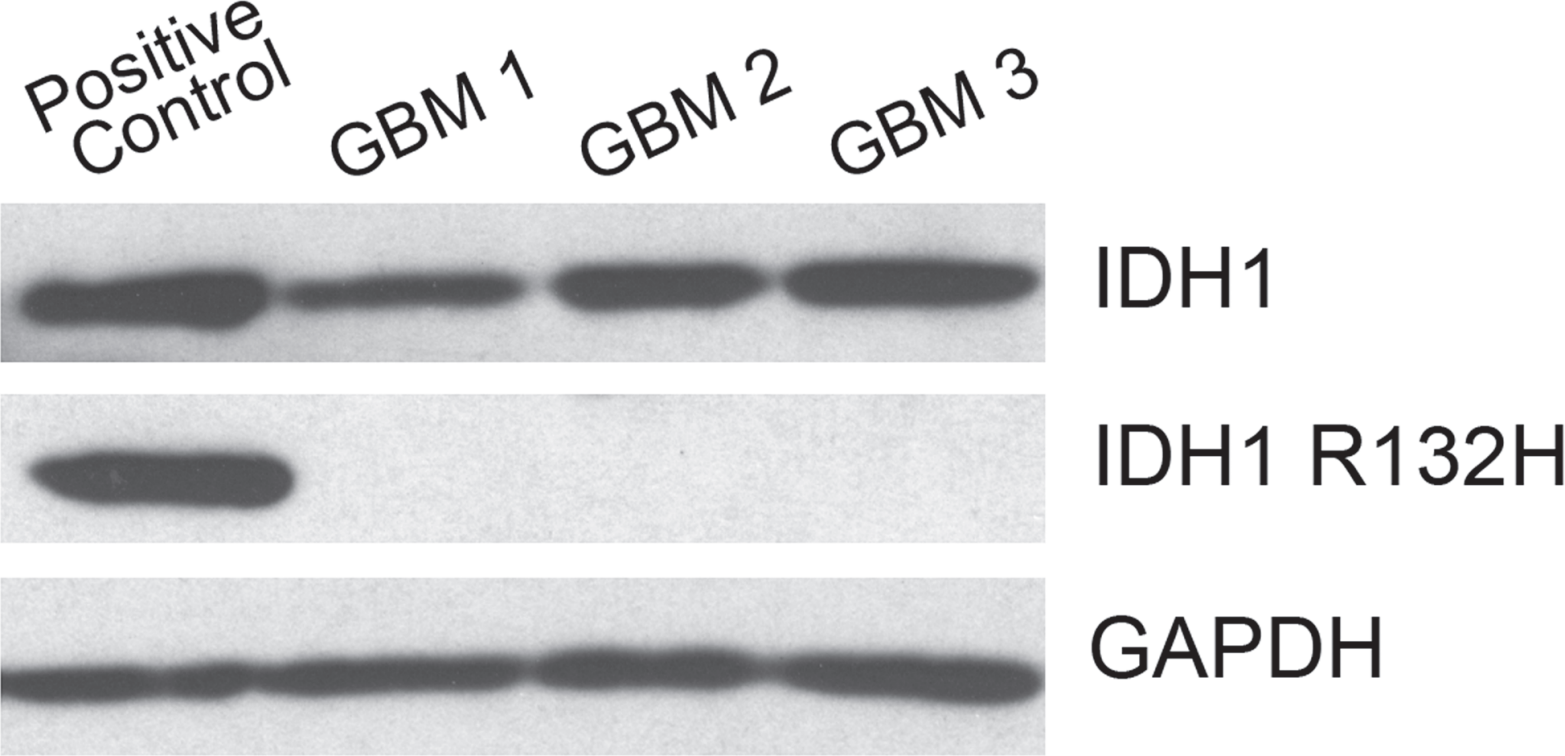
IDH1 mutation status of human GBM samples. Three frozen GBM specimens used for previous analysis and known to express CD97 were found to express wild-type IDH1, but not mutant IDH1 (R132H) by Western blot.

Using patient data from TCGA we sought to characterize the expression of CD97 across genetic subtypes of GBM. CD97 gene expression was quantified using a z-score; tumors with z-scores greater than 1 were defined as upregulated while those with scores less than −1 were defined as downregulated. Across all subtypes, there was a trend of CD97 upregulation among classical and mesenchymal GBMs and CD97 downregulation in the neural and proneural subtypes (Fig. 4). The classical subtype had the highest percentage of CD97 upregulated tumors (59 of 133, 44%) followed by mesenchymal (38 of 150, 25%). Only 9 of 84 neural GBMs (11%) and 3 of 128 proneural GBMs (2%) exhibited upregulation of CD97. The difference in proportion of tumors with CD97 upregulation across all subtypes was statistically significant (p<0.001). The mean z-score in each subtype was as follows: classical 0.99, mesenchymal 0.39, neural −0.18, and proneural −0.72, differences that were statistically significant (p<0.001). Given the importance of IDH1 mutations in the development of secondary GBM, we sought to compare CD97 expression among IDH1 mutant and IDH1 wild-type GBMs. Among patients with IDH1 mutations, 13 of 21 (62%) demonstrated downregulation of CD97, compared to 62 of 479 (13%) wild-type GBMs (p<0.001). The mean z-score among IDH1 wild-type tumors was 0.22 compared to −0.92 among IDH1 mutant tumors (p<0.001). All of the frozen GBM specimens collected at our institution and found to express CD97 were also IDH1 wild-type (Fig. 5).

### CD97 expression in brain tumor initiating cells

**Figure 6:**
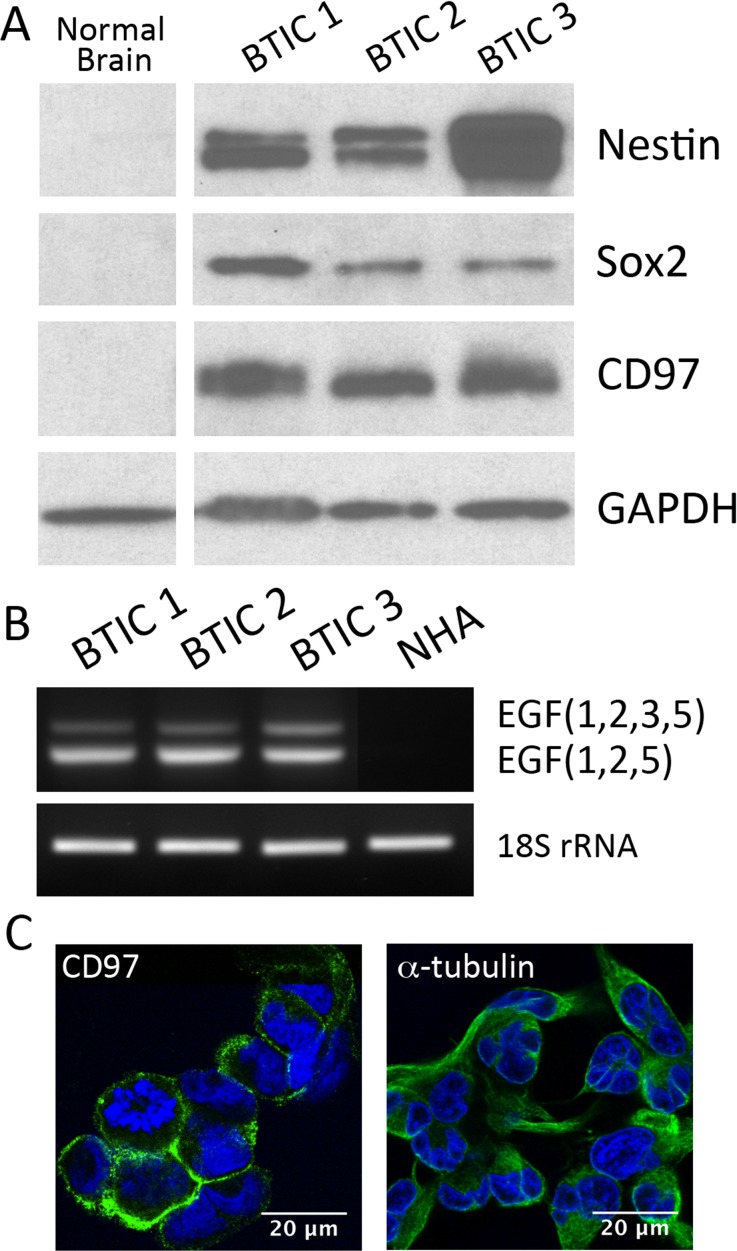
CD97 expression in glioblastoma-derived brain tumor initiating cells. Brain tumor initiating cell lines were derived from three patients with histologically confirmed GBM. These cell lines were found to express the stem cell markers nestin and Sox2, as well as CD97, while normal human brain expressed none of these (A). PCR analysis of these cell lines found them to express the EGF(1,2,5) and EGF(1,2,3,5) isoforms of CD97 as confirmed by sequencing analysis (B). Immunocytochemistry demonstrated localization of CD97 to the cell membrane in BTIC cell line 3 (C).

BTICs are glioma stem-like cells capable of both self-renewal and differentiation. Analysis of CD97 expression in this unique population of cells has not been performed to date. Using a well-validated method for establishing adherent BTICs in culture [27], we generated three unique BTIC lines from patients treated at our institution. All three were found to express the neural stem cell markers nestin and Sox2 (Fig. 6A). Additionally, all cell lines expressed consistently high levels of CD97 protein compared to normal brain. With respect to isoform expression, these BTICs all expressed the same isoforms as established GBM cell lines and frozen tumor specimens (Fig. 6B). Additionally, on immunocytochemistry the CD97 protein in these BTICs was found to localize to the cell membrane (Fig. 6C).

## Discussion

CD97 is a novel GBM antigen implicated in tumor invasion and associated with decreased overall survival. The goal of the present study was to characterize CD97 isoform expression across histologic grades of glioma and within GBM-derived BTICs. We demonstrate CD97 protein expression in both GBM and BTICs, but not in low grade or anaplastic astrocytomas. Furthermore, we shown that only 2 isoforms, EGF(1,2,5) and EGF(1,2,3,5), are expressed in these tumors. Using data acquired from TCGA, we show that GBMs with increased expression of CD97 are almost exclusively of the classical or mesenchymal subtype. Furthermore, tumors with IDH1 mutations had significantly lower CD97 expression, suggesting a less invasive phenotype in these so-called secondary GBMs, which are believed to originate from low grade lesions that undergo malignant transformation.

The identification of the EGF(1,2,5) isoform in GBM and BTICs is significant since this isoform has been shown to support local tumor growth, enhance migration, and promote metastatic spread in multiple cancer types [19,30]. The EGF(1–5) and EGF(1,2,5) isoforms bind chondroitin sulfate and CD55, respectively. Since the fourth EGF domain is required for binding of chondroitin sulfate, EGF(1,2,5) and EGF(1,2,3,5) possess similar ligand binding properties [31]. Immunohistochemical studies in gastric and colorectal carcinoma have shown that CD97 and CD55 are localized to the tumor’s invasive front and correlate with both TNM stage and survival, further implicating EGF(1,2,5) expression as an important prognostic factor [32–35]. The EGF(1–5) isoform, through coengagement of the integrin α5β1 and chondroitin sulfate, has been shown to stimulate migration and invasion of human umbilical vein endothelial cells (HUVECs). Tumors with this isoform were found to possess increased microvessel density compared to those containing the EGF(1,2,5) isoform, however this is not believed to result in differential tumor growth rates [11,19]. Both EGF(1,2,5) and EGF(1–5) are capable binding the integrin α5β1; the β1 subunit has been shown to play an important role in the malignant behavior and invasive phenotype associated with gliomas [13,36,37]. Integrins are attractive therapeutic targets for patients with GBM and Cilengitide, a peptide targeting the RGD-motif, has shown modest efficacy and is currently under investigation in a Phase III clinical trial for patients with newly diagnosed GBM (NCT00689221) [38,39]. We show that the proportion of CD97 isoforms in gliomas is essentially constant with 15% EGF(1,2,3,5) and 85% and EGF(1,2,5) in all samples. One may have expected to observe a proportionate increase in EGF(1,2,5) with tumor grade, particularly given its established role in tumor invasion, however it appears that total CD97, not specific isoform expression, is a more important predictor of tumor aggression. The increase in EGF(1,2,3,5) compared to normal human astrocytes suggests that the shift in isoform proportion may occur early in gliomagenesis or represent a consequence of other genetic aberrations found in gliomas but not normal glia.

In this study we present the first characterization of CD97 expression in BTICs. These cells, which possess the capacity for both self-renewal and differentiation, play important roles in chemoresistance [21], radioresistance [22], angiogenesis [23], and tumor recurrence [24–26]. Although there is increasing evidence that these cells play an important role in gliomagenesis, there is limited data characterizing their invasive potential [40–42]. Experiments performed primarily in vitro suggest that glioma stem cells and BTICs may possess increased invasive potential compared to differentiated glioma cells, although there is also evidence that cancer stem cells may adopt a less invasive phenotype in favor of one that is more proliferative [43–46]. The identification of CD97 in our BTIC lines provides a potential mechanism for their invasive phenotype. These findings, along with data showing similar proportions of isoforms among patient-matched GBMs and GBM-derived BTICs, suggest that although a shift in isoform expression may occur early in gliomagenesis, upregulation of CD97 occurs later and is perhaps unique to de novo GBMs compared to low grade astrocytomas or secondary GBMs. These data also suggest that although CD97 can confer increased invasiveness in glioma cells, it is likely not responsible for observed differences in invasive potential between BTICs and GBM cells. Lastly, given our data demonstrating an increase in the proportion of EGF(1,2,3,5) isoform among astrocytomas compared to normal human astrocytes, there is a possibility that this shift in expression patterns can be exploited for diagnostic purposes in certain clinical scenarios where there is uncertainty as to the identify of tissues, for example in differentiating possible recurrent tumor from normal adjacent brain with treatment effect.

Although limited by sample size, our analysis of TCGA data provides additional insight towards the role of CD97 in GBM. The stratification of GBM into four distinct molecular subtypes has drawn attention to the complex heterogeneity of this disease[47–51]. The classical and mesenchymal subtypes are generally associated with worse overall survival, and given our data showing increased CD97 expression in these subgroups, the survival discrepancy may be explained by increased tumor invasion. Additionally, our data demonstrating significantly decreased expression of CD97 among IDH1 mutant GBMs suggest that CD97 may be an important mediator of invasion in de novo or primary GBMs, but not secondary GBMs. Studies have shown that IDH1 mutation status is inversely related to tumor grade, with higher mutation rates in low grade astrocytomas compared to GBMs [52–59]. Furthermore, IDH1 mutations are significantly more common in secondary GBMs, likely because these tumors originated from low grade lesions [58,59]. Additional studies are needed to determine if CD97 upregulation is significantly associated with either de novo or secondary GBMs, however since we did not detect CD97 in our low grade astrocytomas, we suspect CD97 may be a marker of primary GBM, particularly given its expression in BTICs.

There are certain limitations to the present study. Although we were able to characterize isoform expression at the transcript level, commercially available antibodies cannot distinguish the EGF(1,2,5) and EGF(1,2,3,5) isoforms. However, by virtue of lacking the fourth EGF domain, the EGF(1,2,5) and EGF(1,2,3,5) isoforms exhibit similar ligand binding properties, with affinity for CD55 and the integrin α5β1 [7,11]. In a previous study we demonstrate that CD97 confers an invasive phenotype and is associated with decreased survival in GBM patients [4]. Although we do not present functional data demonstrating unique roles for these isoforms in low grade gliomas or BTICs, we suspect that they perform essentially identical functions given that GBM specimens and BTICs express the same isoforms used in our previous study. The downstream signaling pathways associated with CD97 have not yet been fully elucidated, there is evidence that these receptors signal through Gα12/13, resulting in increased RHO-GTP and activation of extracellular signal-regulated kinase (ERK) in prostate and thyroid cancers [60,61]. The authors also showed that CD97 heterodimerizes and positively regulates lysophosphatidic acid receptor 1 (LPAR1) signaling, a well-established mediator of tumorigenesis and metastasis in prostate cancer [62]. Our laboratory is currently investigating the potential significance of these relationships in GBM.

Although several markers of tumor proliferation and metabolism have been identified in GBM and are used clinically (EGFRvIII amplification, MGMT promoter methylation, IDH1 mutation status), prognostic markers of invasive capacity are largely lacking. Such markers can offer improved prognostic capabilities and contribute to the identification of novel therapeutic targets. In this study we identify the specific isoforms of CD97, a novel pro-invasive glioma antigen, across histologic grades of glioma and within BTICs. We also demonstrate a trend towards increased CD97 expression among the classical and mesenchymal GBM subtypes, as well as decreased expression among IDH1 mutant tumors.

## Conclusions

CD97 confers an invasive phenotype and is associated with decreased survival in GBM patients. We show that the short isoform of this protein is expressed in both GBM and BTICs, but not low grade or anaplastic astrocytomas, providing a potential mechanism for the invasive nature of these tumors. We also show that upregulation of CD97 is more common among the classical and mesenchymal subtype of GBM, suggesting that the poor prognosis associated with these specific tumors is related to a more invasive phenotype.

## References

[1] Stupp R, Mason WP, van den Bent MJ, Weller M, Fisher B, et al (2005) Radiotherapy plus concomitant and adjuvant temozolomide for glioblastoma. N Engl J Med 352: 987–996. 15758009 10.1056/NEJMoa043330

[2] Laws ER, Parney IF, Huang W, Anderson F, Morris AM, et al (2003) Survival following surgery and prognostic factors for recently diagnosed malignant glioma: data from the Glioma Outcomes Project. J Neurosurg 99: 467–473. 12959431 10.3171/jns.2003.99.3.0467

[3] Parsa AT, Wachhorst S, Lamborn KR, Prados MD, McDermott MW, et al (2005) Prognostic significance of intracranial dissemination of glioblastoma multiforme in adults. J Neurosurg 102: 622–628. 15871503 10.3171/jns.2005.102.4.0622

[4] Safaee M, Clark AJ, Oh MC, Ivan ME, Bloch O, et al (2013) Overexpression of CD97 Confers an Invasive Phenotype in Glioblastoma Cells and Is Associated with Decreased Survival of Glioblastoma Patients. PLoS One 8: e62765 10.1371/journal.pone.0062765 23658650 PMC3637305

[5] Yona S, Lin HH, Siu WO, Gordon S, Stacey M (2008) Adhesion-GPCRs: emerging roles for novel receptors. Trends Biochem Sci 33: 491–500. 10.1016/j.tibs.2008.07.005 18789697

[6] Eichler W, Hamann J, Aust G (1997) Expression characteristics of the human CD97 antigen. Tissue Antigens 50: 429–438. 9389316 10.1111/j.1399-0039.1997.tb02897.x

[7] Hamann J, Vogel B, van Schijndel GM, van Lier RA (1996) The seven-span transmembrane receptor CD97 has a cellular ligand (CD55, DAF). J Exp Med 184: 1185–1189. 9064337 10.1084/jem.184.3.1185 PMC2192782

[8] Stacey M, Chang GW, Davies JQ, Kwakkenbos MJ, Sanderson RD, et al (2003) The epidermal growth factor-like domains of the human EMR2 receptor mediate cell attachment through chondroitin sulfate glycosaminoglycans. Blood 102: 2916–2924. 12829604 10.1182/blood-2002-11-3540

[9] Hamann J, Stortelers C, Kiss-Toth E, Vogel B, Eichler W, et al (1998) Characterization of the CD55 (DAF)-binding site on the seven-span transmembrane receptor CD97. Eur J Immunol 28: 1701–1707. 9603477 10.1002/(SICI)1521-4141(199805)28:05<1701::AID-IMMU1701>3.0.CO;2-2

[10] Qian YM, Haino M, Kelly K, Song WC (1999) Structural characterization of mouse CD97 and study of its specific interaction with the murine decay-accelerating factor (DAF, CD55). Immunology 98: 303–311. 10540231 10.1046/j.1365-2567.1999.00859.x PMC2326925

[11] Wang T, Ward Y, Tian L, Lake R, Guedez L, et al (2005) CD97, an adhesion receptor on inflammatory cells, stimulates angiogenesis through binding integrin counterreceptors on endothelial cells. Blood 105: 2836–2844. 15576472 10.1182/blood-2004-07-2878

[12] Tchaicha JH, Reyes SB, Shin J, Hossain MG, Lang FF, et al (2011) Glioblastoma angiogenesis and tumor cell invasiveness are differentially regulated by beta8 integrin. Cancer Res 71: 6371–6381. 10.1158/0008-5472.CAN-11-0991 21859829 PMC3193578

[13] Friedlander DR, Zagzag D, Shiff B, Cohen H, Allen JC, et al (1996) Migration of brain tumor cells on extracellular matrix proteins in vitro correlates with tumor type and grade and involves alphaV and beta1 integrins. Cancer Res 56: 1939–1947. 8620517

[14] Schrappe M, Klier FG, Spiro RC, Waltz TA, Reisfeld RA, et al (1991) Correlation of chondroitin sulfate proteoglycan expression on proliferating brain capillary endothelial cells with the malignant phenotype of astroglial cells. Cancer Res 51: 4986–4993. 1893386

[15] Steinert M, Wobus M, Boltze C, Schutz A, Wahlbuhl M, et al (2002) Expression and regulation of CD97 in colorectal carcinoma cell lines and tumor tissues. Am J Pathol 161: 1657–1667. 12414513 10.1016/S0002-9440(10)64443-4 PMC1850798

[16] Holting T, Siperstein AE, Clark OH, Duh QY (1995) Epidermal growth factor (EGF)- and transforming growth factor alpha-stimulated invasion and growth of follicular thyroid cancer cells can be blocked by antagonism to the EGF receptor and tyrosine kinase in vitro. Eur J Endocrinol 132: 229–235. 7858744 10.1530/eje.0.1320229

[17] Aust G, Steinert M, Schutz A, Boltze C, Wahlbuhl M, et al (2002) CD97, but not its closely related EGF-TM7 family member EMR2, is expressed on gastric, pancreatic, and esophageal carcinomas. Am J Clin Pathol 118: 699–707. 12428789 10.1309/A6AB-VF3F-7M88-C0EJ

[18] Aust G, Eichler W, Laue S, Lehmann I, Heldin NE, et al (1997) CD97: a dedifferentiation marker in human thyroid carcinomas. Cancer Res 57: 1798–1806. 9135025

[19] Galle J, Sittig D, Hanisch I, Wobus M, Wandel E, et al (2006) Individual cell-based models of tumor-environment interactions: Multiple effects of CD97 on tumor invasion. Am J Pathol 169: 1802–1811. 17071601 10.2353/ajpath.2006.060006 PMC1780199

[20] Chidambaram A, Fillmore HL, Van Meter TE, Dumur CI, Broaddus WC (2012) Novel report of expression and function of CD97 in malignant gliomas: correlation with Wilms tumor 1 expression and glioma cell invasiveness. J Neurosurg 116: 843–853. 10.3171/2011.11.JNS111455 22313360

[21] Liu G, Yuan X, Zeng Z, Tunici P, Ng H, et al (2006) Analysis of gene expression and chemoresistance of CD133+ cancer stem cells in glioblastoma. Mol Cancer 5: 67 17140455 10.1186/1476-4598-5-67 PMC1697823

[22] Bao S, Wu Q, McLendon RE, Hao Y, Shi Q, et al (2006) Glioma stem cells promote radioresistance by preferential activation of the DNA damage response. Nature 444: 756–760. 17051156 10.1038/nature05236

[23] Bao S, Wu Q, Sathornsumetee S, Hao Y, Li Z, et al (2006) Stem cell-like glioma cells promote tumor angiogenesis through vascular endothelial growth factor. Cancer Res 66: 7843–7848. 16912155 10.1158/0008-5472.CAN-06-1010

[24] Beier D, Hau P, Proescholdt M, Lohmeier A, Wischhusen J, et al (2007) CD133(+) and CD133(-) glioblastoma-derived cancer stem cells show differential growth characteristics and molecular profiles. Cancer Res 67: 4010–4015. 17483311 10.1158/0008-5472.CAN-06-4180

[25] Tamase A, Muraguchi T, Naka K, Tanaka S, Kinoshita M, et al (2009) Identification of tumor-initiating cells in a highly aggressive brain tumor using promoter activity of nucleostemin. Proc Natl Acad Sci U S A 106: 17163–17168. 10.1073/pnas.0905016106 19805150 PMC2761321

[26] Chen J, Li Y, Yu TS, McKay RM, Burns DK, et al (2012) A restricted cell population propagates glioblastoma growth after chemotherapy. Nature 488: 522–526. 10.1038/nature11287 22854781 PMC3427400

[27] Pollard SM, Yoshikawa K, Clarke ID, Danovi D, Stricker S, et al (2009) Glioma stem cell lines expanded in adherent culture have tumor-specific phenotypes and are suitable for chemical and genetic screens. Cell Stem Cell 4: 568–580. 10.1016/j.stem.2009.03.014 19497285

[28] Sun Y, Pollard S, Conti L, Toselli M, Biella G, et al (2008) Long-term tripotent differentiation capacity of human neural stem (NS) cells in adherent culture. Mol Cell Neurosci 38: 245–258. 10.1016/j.mcn.2008.02.014 18450476

[29] Brosseau JP, Lucier JF, Lapointe E, Durand M, Gendron D, et al (2010) High-throughput quantification of splicing isoforms. RNA 16: 442–449. 10.1261/rna.1877010 20038630 PMC2811672

[30] Liu D, Trojanowicz B, Ye L, Li C, Zhang L, et al (2012) The invasion and metastasis promotion role of CD97 small isoform in gastric carcinoma. PLoS One 7: e39989 10.1371/journal.pone.0039989 22768192 PMC3386904

[31] Lin HH, Stacey M, Saxby C, Knott V, Chaudhry Y, et al (2001) Molecular analysis of the epidermal growth factor-like short consensus repeat domain-mediated protein-protein interactions: dissection of the CD97-CD55 complex. J Biol Chem 276: 24160–24169. 11297558 10.1074/jbc.M101770200

[32] Durrant LG, Chapman MA, Buckley DJ, Spendlove I, Robins RA, et al (2003) Enhanced expression of the complement regulatory protein CD55 predicts a poor prognosis in colorectal cancer patients. Cancer Immunol Immunother 52: 638–642. 12811528 10.1007/s00262-003-0402-y PMC11034327

[33] Liu Y, Chen L, Peng SY, Chen ZX, Hoang-Vu C (2005) Role of CD97(stalk) and CD55 as molecular markers for prognosis and therapy of gastric carcinoma patients. J Zhejiang Univ Sci B 6: 913–918. 16130195 10.1631/jzus.2005.B0913 PMC1389911

[34] Liu Y, Chen L, Peng S, Chen Z, Gimm O, et al (2005) The expression of CD97EGF and its ligand CD55 on marginal epithelium is related to higher stage and depth of tumor invasion of gastric carcinomas. Oncol Rep 14: 1413–1420. 16273233

[35] Wu J, Lei L, Wang S, Gu D, Zhang J (2012) Immunohistochemical expression and prognostic value of CD97 and its ligand CD55 in primary gallbladder carcinoma. J Biomed Biotechnol 2012: 587672 10.1155/2012/587672 22547928 PMC3324160

[36] Tonn JC, Wunderlich S, Kerkau S, Klein CE, Roosen K (1998) Invasive behaviour of human gliomas is mediated by interindividually different integrin patterns. Anticancer Res 18: 2599–2605. 9703915

[37] Rooprai HK, Vanmeter T, Panou C, Schnull S, Trillo-Pazos G, et al (1999) The role of integrin receptors in aspects of glioma invasion in vitro. Int J Dev Neurosci 17: 613–623. 10571422 10.1016/s0736-5748(99)00051-9

[38] Gilbert MR, Kuhn J, Lamborn KR, Lieberman F, Wen PY, et al (2012) Cilengitide in patients with recurrent glioblastoma: the results of NABTC 03–02, a phase II trial with measures of treatment delivery. J Neurooncol 106: 147–153. 10.1007/s11060-011-0650-1 21739168 PMC4351869

[39] Scaringi C, Minniti G, Caporello P, Enrici RM (2012) Integrin inhibitor cilengitide for the treatment of glioblastoma: a brief overview of current clinical results. Anticancer Res 32: 4213–4223. 23060541

[40] Singh SK, Hawkins C, Clarke ID, Squire JA, Bayani J, et al (2004) Identification of human brain tumour initiating cells. Nature 432: 396–401. 15549107 10.1038/nature03128

[41] Galli R, Binda E, Orfanelli U, Cipelletti B, Gritti A, et al (2004) Isolation and characterization of tumorigenic, stem-like neural precursors from human glioblastoma. Cancer Res 64: 7011–7021. 15466194 10.1158/0008-5472.CAN-04-1364

[42] Lee J, Kotliarova S, Kotliarov Y, Li A, Su Q, et al (2006) Tumor stem cells derived from glioblastomas cultured in bFGF and EGF more closely mirror the phenotype and genotype of primary tumors than do serum-cultured cell lines. Cancer Cell 9: 391–403. 16697959 10.1016/j.ccr.2006.03.030

[43] Yu SP, Yang XJ, Zhang B, Ming HL, Chen C, et al (2011) Enhanced invasion in vitro and the distribution patterns in vivo of CD133+ glioma stem cells. Chin Med J (Engl) 124: 2599–2604. 22040410

[44] Cheng L, Wu Q, Guryanova OA, Huang Z, Huang Q, et al (2011) Elevated invasive potential of glioblastoma stem cells. Biochem Biophys Res Commun 406: 643–648. 10.1016/j.bbrc.2011.02.123 21371437 PMC3065536

[45] Qiu B, Zhang D, Tao J, Tie X, Wu A, et al (2012) Human brain glioma stem cells are more invasive than their differentiated progeny cells in vitro. J Clin Neurosci 19: 130–134. 10.1016/j.jocn.2011.06.014 22153826

[46] Biddle A, Liang X, Gammon L, Fazil B, Harper LJ, et al (2011) Cancer stem cells in squamous cell carcinoma switch between two distinct phenotypes that are preferentially migratory or proliferative. Cancer Res 71: 5317–5326. 10.1158/0008-5472.CAN-11-1059 21685475

[47] Cancer Genome Atlas Research N (2008) Comprehensive genomic characterization defines human glioblastoma genes and core pathways. Nature 455: 1061–1068. 10.1038/nature07385 18772890 PMC2671642

[48] Verhaak RG, Hoadley KA, Purdom E, Wang V, Qi Y, et al (2010) Integrated genomic analysis identifies clinically relevant subtypes of glioblastoma characterized by abnormalities in PDGFRA, IDH1, EGFR, and NF1. Cancer Cell 17: 98–110. 10.1016/j.ccr.2009.12.020 20129251 PMC2818769

[49] Tso CL, Freije WA, Day A, Chen Z, Merriman B, et al (2006) Distinct transcription profiles of primary and secondary glioblastoma subgroups. Cancer Res 66: 159–167. 16397228 10.1158/0008-5472.CAN-05-0077

[50] Beroukhim R, Getz G, Nghiemphu L, Barretina J, Hsueh T, et al (2007) Assessing the significance of chromosomal aberrations in cancer: methodology and application to glioma. Proc Natl Acad Sci U S A 104: 20007–20012. 18077431 10.1073/pnas.0710052104 PMC2148413

[51] Phillips HS, Kharbanda S, Chen R, Forrest WF, Soriano RH, et al (2006) Molecular subclasses of high-grade glioma predict prognosis, delineate a pattern of disease progression, and resemble stages in neurogenesis. Cancer Cell 9: 157–173. 16530701 10.1016/j.ccr.2006.02.019

[52] Balss J, Meyer J, Mueller W, Korshunov A, Hartmann C, et al (2008) Analysis of the IDH1 codon 132 mutation in brain tumors. Acta Neuropathol 116: 597–602. 10.1007/s00401-008-0455-2 18985363

[53] Bleeker FE, Atai NA, Lamba S, Jonker A, Rijkeboer D, et al (2010) The prognostic IDH1 (R132) mutation is associated with reduced NADP+-dependent IDH activity in glioblastoma. Acta Neuropathol 119: 487–494. 10.1007/s00401-010-0645-6 20127344 PMC2841753

[54] Hartmann C, Meyer J, Balss J, Capper D, Mueller W, et al (2009) Type and frequency of IDH1 and IDH2 mutations are related to astrocytic and oligodendroglial differentiation and age: a study of 1,010 diffuse gliomas. Acta Neuropathol 118: 469–474. 10.1007/s00401-009-0561-9 19554337

[55] Sanson M, Marie Y, Paris S, Idbaih A, Laffaire J, et al (2009) Isocitrate dehydrogenase 1 codon 132 mutation is an important prognostic biomarker in gliomas. J Clin Oncol 27: 4150–4154. 10.1200/JCO.2009.21.9832 19636000

[56] Watanabe T, Nobusawa S, Kleihues P, Ohgaki H (2009) IDH1 mutations are early events in the development of astrocytomas and oligodendrogliomas. Am J Pathol 174: 1149–1153. 10.2353/ajpath.2009.080958 19246647 PMC2671348

[57] Yan H, Parsons DW, Jin G, McLendon R, Rasheed BA, et al (2009) IDH1 and IDH2 mutations in gliomas. N Engl J Med 360: 765–773. 10.1056/NEJMoa0808710 19228619 PMC2820383

[58] Ichimura K, Pearson DM, Kocialkowski S, Backlund LM, Chan R, et al (2009) IDH1 mutations are present in the majority of common adult gliomas but rare in primary glioblastomas. Neuro Oncol 11: 341–347. 10.1215/15228517-2009-025 19435942 PMC2743214

[59] Nobusawa S, Watanabe T, Kleihues P, Ohgaki H (2009) IDH1 mutations as molecular signature and predictive factor of secondary glioblastomas. Clin Cancer Res 15: 6002–6007. 10.1158/1078-0432.CCR-09-0715 19755387

[60] Ward Y, Lake R, Yin JJ, Heger CD, Raffeld M, et al (2011) LPA receptor heterodimerizes with CD97 to amplify LPA-initiated RHO-dependent signaling and invasion in prostate cancer cells. Cancer Res 71: 7301–7311. 10.1158/0008-5472.CAN-11-2381 21978933 PMC6697138

[61] Ward Y, Lake R, Martin PL, Killian K, Salerno P, et al. (2012) CD97 amplifies LPA receptor signaling and promotes thyroid cancer progression in a mouse model. Oncogene. 10.1038/onc.2012.301 PMC7561260 22797060

[62] Lin ME, Herr DR, Chun J (2010) Lysophosphatidic acid (LPA) receptors: signaling properties and disease relevance. Prostaglandins Other Lipid Mediat 91: 130–138. 10.1016/j.prostaglandins.2009.02.002 20331961 PMC2845529

